# Connective Tissue Growth Factor Is Related to All-cause Mortality in Hemodialysis Patients and Is Lowered by On-line Hemodiafiltration: Results from the Convective Transport Study

**DOI:** 10.3390/toxins11050268

**Published:** 2019-05-13

**Authors:** Claire H. den Hoedt, Maaike K. van Gelder, Muriel P. Grooteman, Menso J. Nubé, Peter J. Blankestijn, Roel Goldschmeding, Robbert Jan Kok, Michiel L. Bots, Marinus A. van den Dorpel, Karin G. F. Gerritsen

**Affiliations:** 1 Department of Nephrology and Hypertension, University Medical Center Utrecht, Utrecht 3584 CX, The Netherlands; c.h.denhoedt@erasmusmc.nl (C.H.d.H.); m.k.vangelder-5@umcutrecht.nl (M.K.v.G.); P.J.Blankestijn@umcutrecht.nl (P.J.B.); 2 Department of Internal Medicine, Maasstad Hospital, Rotterdam 3079 DZ, The Netherlands; DorpelM@maasstadziekenhuis.nl; 3 Amsterdam UMC, Vrije Universiteit Amsterdam, Amsterdam Cardiovascular Sciences, Nephrology, Amsterdam 1081 HV, The Netherlands; mpc.grooteman@vumc.nl (M.P.G.); m.nube@spaarnegasthuis.nl (M.J.N.); 4 Department of Pathology, University Medical Center Utrecht, Utrecht 3584 CX, The Netherlands; R.Goldschmeding@umcutrecht.nl; 5 Department of Pharmaceutics, Utrecht Institute for Pharmaceutical Sciences, Utrecht 3584 CG, The Netherlands; R.J.Kok@uu.nl; 6 Julius Center for Health Sciences and Primary Care, University Medical Center Utrecht, University Utrecht, 3508 GA Utrecht, The Netherlands; M.L.Bots@umcutrecht.nl

**Keywords:** Connective tissue growth factor, CCN intercellular signalling proteins, haemodialysis, haemodiafiltration, convection, end stage kidney disease, cardiovascular disease, mortality

## Abstract

Connective tissue growth factor (CTGF) plays a key role in the pathogenesis of tissue fibrosis. The aminoterminal fragment of CTGF is a middle molecule that accumulates in chronic kidney disease. The aims of this study are to explore determinants of plasma CTGF in hemodialysis (HD) patients, investigate whether CTGF relates to all-cause mortality in HD patients, and investigate whether online-hemodiafiltration (HDF) lowers CTGF. Data from 404 patients participating in the CONvective TRAnsport STudy (CONTRAST) were analyzed. Patients were randomized to low-flux HD or HDF. Pre-dialysis CTGF was measured by sandwich ELISA at baseline, after six and 12 months. CTGF was inversely related in multivariable analysis to glomerular filtration rate (GFR) (*p* < 0.001) and positively to cardiovascular disease (CVD) (*p* = 0.006), dialysis vintage (*p* < 0.001), interleukin-6 (*p* < 0.001), beta-2-microglobulin (*p* = 0.045), polycystic kidney disease (*p* < 0.001), tubulointerstitial nephritis (*p* = 0.002), and renal vascular disease (*p* = 0.041). Patients in the highest quartile had a higher mortality risk compared to those in the lowest quartile (HR 1.7, 95% CI: 1.02–2.88, *p* = 0.043). HDF lowered CTGF with 4.8% between baseline and six months, whereas during HD, CTGF increased with 4.9% (*p* < 0.001). In conclusion, in HD patients, CTGF is related to GFR, CVD and underlying renal disease and increased the risk of all-cause mortality. HDF reduces CTGF.

## 1. Introduction

Connective tissue growth factor (CTGF/CCN2, Molecular weight (Mw) 38 kilo Dalton (kDa)) is a profibrotic growth factor that plays a key role in the pathogenesis of tissue fibrosis [1]. CTGF is expressed by many cell types, including endothelial cells, vascular smooth muscle cells, fibroblasts, cardiac myocytes, and renal tubular epithelial cells [2]. Upregulation of CTGF is caused by several factors, such as angiotensin II, oxidative stress, advanced glycation end products, and cell stretch, conditions that are highly prevalent in hemodialysis (HD) patients [3,4,5]. The proteolytic aminoterminal fragment of CTGF (N-CTGF, Mw 18 kD) is the predominant form of CTGF in plasma. Plasma (N-)CTGF is exclusively eliminated by the kidneys, accumulates in chronic kidney disease, with highest levels in end stage kidney disease (ESKD) [6,7].

CTGF has been reported to be involved in various chronic diseases including systemic sclerosis, lung fibrosis, cardiac fibrosis, atherosclerosis, liver fibrosis, kidney fibrosis, diabetic nephropathy (DN), and peritoneal fibrosis in peritoneal dialysis patients [1,2]. CTGF is considered an important regulator of cardiac fibrosis and post-ischemic cardiac remodelling, which may lead to diastolic heart failure [3,8,9,10,11,12,13]. In addition, CTGF is highly expressed in atherosclerotic plaques and has been implicated in atherogenesis [14,15,16]. In a study among 1227 patients with atherosclerotic disease, plasma CTGF was independently related to an increased risk of cardiovascular events and mortality [17]. Furthermore, in a study among 1050 patients with type 1 DM, elevated concentrations of CTGF were associated with an increased common carotid intima media thickness, hypertension and presence of macroalbuminuria [18]. In a study of 198 type I diabetics with diabetic nephropathy, elevated concentrations of CTGF were related to an increased risk of progression to ESKD, and to death from any cause within 12 years [19].

The relationship between CTGF and CVD, an important predictor of mortality, suggests that CTGF might play a role in excess cardiovascular morbidity and mortality in HD patients. Whether plasma CTGF is related to clinical outcome and whether treatment with HDF results in a sustained decrease of plasma CTGF is unknown. Previously, we reported that plasma CTGF was effectively reduced by a single HDF session, but not by low flux HD [6].

The first aim of the present study was to explore the determinants of plasma CTGF in a large cohort of patients on conventional low-flux HD. The second aim was to investigate whether CTGF is related to all-cause mortality and cardiovascular morbidity and mortality in HD patients. The third aim was to compare the difference in pre-dialysis CTGF concentrations after 6 and 12 months of treatment between low flux HD and HDF.

## 2. Results

### 2.1. Patient and Dialysis Characteristics

The characteristics of the patients are summarized in Table 1. Mean age of patients (*n* = 404) was 64 ± 14 years and 62% was male. Median CTGF concentration was 3.5 (2.7–4.3) nmol/L. Patients had the following underlying renal diseases: renal vascular disease 129 (32%), DM 64 (16%), primary glomerulonephritis 40 (10%), interstitial nephropathy 38 (9%), PKD 28 (7%), multisystem disease 18 (5%), other 38 (9%), or unknown 49 (12%). The majority of patients (93%) received dialysis treatment three times per week. 

### 2.2. Determinants of Plasma CTGF 

The association between CTGF and various factors are presented in Table 2. CTGF was inversely related to eGFR (*p* < 0.001), and with borderline significance to use of RAAS blockers (*p* = 0.05). CTGF related positively to previous CVD (*p* = 0.006), dialysis vintage (*p* < 0.001) and IL-6 (*p* < 0.001) and with borderline significance to B2M (*p* = 0.05) and pulse pressure (*p* = 0.06). In addition, CTGF was higher in patients who had underlying PKD (*p* < 0.001), TIN (*p* = 0.002) or renal vascular disease (*p* = 0.04), as compared to patients with unknown underlying renal diseases, used as the reference group.

When previous CVD was replaced in the model by previous coronary heart disease, cerebrovascular disease, or PVD, it appeared that the relation between CTGF and previous CVD was most evident in PVD (β = 0.20, *p* < 0.001, 22.5% (11.9%–34.2%) increase in CTGF if previous PVD was present).

### 2.3. CTGF and All-cause Mortality 

After a median follow-up of 3.1 (range 0.1 to 6.6) years, 158 patients (39%) had died. Baseline CTGF concentrations related to mortality, with a hazard ratio (HR) of 1.10 (95% CI, 1.01–1.19, *p* = 0.02) for every 1 nmol/L increase in CTGF. After adjustment for confounders, the HR remained 1.09 (95% CI, 1.00 to 1.19, *p* = 0.05). After additional adjustment for CRP and serum albumin, the HR lost its statistical significance (HR 1.09, [95% CI, 0.99–1.19], *p* = 0.07) (*n* = 149 deaths, because of missing data) (Table 3). The ‘on-treatment’ analysis did not change these results (HR for all-cause mortality 1.09; [95% CI, 1.00–1.20], *p* = 0.06, (model including CRP and albumin), 128 deaths).

When quartiles of CTGF were used in the model, the HR of the mortality risk in patients within the highest quartile (CTGF > 4.3 nmol/L) was 1.71 (95% CI, 1.02–2.88, *p* = 0.04), as compared to the patients within the lowest quartile of CTGF, after adjustment for potential confounders (Table 4).

### 2.4. CTGF and Fatal- and Non-fatal Cardiovascular Events and Infectious Mortality 

A fatal or non-fatal cardiovascular event occurred in 131 patients (32%) during a median follow-up of 2.6 (range 0.01 to 6.6) years. The crude HR for cardiovascular events was 1.06 for each increase of plasma CTGF with 1 nmol/L (95% CI, 0.96–1.16, *p* = 0.24). After adjustment, the HR was 1.03 (95% CI, 0.94–1.14, *p* = 0.51) (Table 3). When quartiles of CTGF were used in the model, no consistent relation was observed (Table 4). 

Fifty patients (12%) died of a cardiovascular cause. The crude HR for cardiovascular mortality was 0.99 (95% CI, 0.84–1.18, *p* = 0.93), which did not change substantially after adjustment for potential confounders (HR 1.01 [95% CI, 0.85–1.19], *p* = 0.96) (Table 3). 

Forty patients (10%) died of an infectious cause. The crude HR for infectious mortality was 1.14 (95% CI, 0.99–1.31, *p* = 0.08). After adjustment, the HR was 1.06 (95% CI, 0.92–1.23, *p* = 0.42) (Table 3). 

### 2.5. CTGF Change over Time, HDF versus HD 

Overall, patient characteristics at baseline were balanced between both treatment groups (Table 1), although pre-dialysis B2M and creatinine plasma concentrations were somewhat lower in the HDF group. Median CTGF at baseline was 3.5 (IQR 2.8–4.4) nmol/L in HD patients and 3.5 (IQR 2.7–4.3) nmol/L in HDF patients. During follow-up, Kt/V_urea_ and dialyzer blood flow rate increased in HDF patients, while these parameters remained stable during in HD patients (results not shown). 

**Figure 1:**
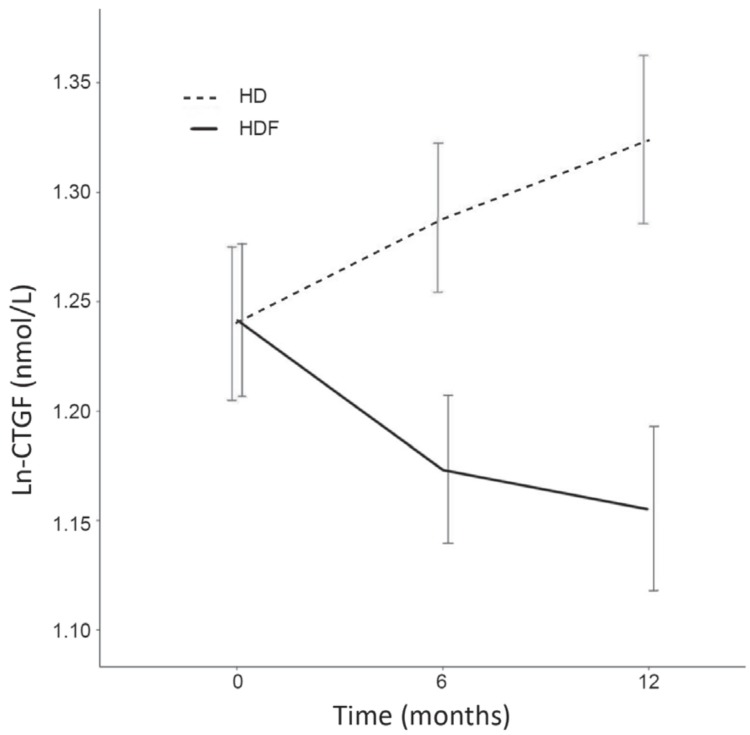
Change in plasma Ln-CTGF (natural logarithm of CTGF) over time, analyzed with complete case analysis (n = 253), expressed as means ± SE.

At the six-month visit, 343 samples were available (176 on HD and 167 on HDF) and at the 12-month visit, 266 samples (133 on HD and 133 on HDF). Changes in CTGF over time were not linear for the HDF patients: after six months, the rate of change seemed to attenuate (Figure 1). Therefore, analyses were stratified by follow up time: Baseline to six months and six to twelve months. The estimates from the linear mixed effect models are presented in Table 5. Between baseline and six months, Ln-CTGF increased with 0.047 nmol/L per 6 months in the HD group (*p* = 0.007), and decreased with 0.050 nmol/L per 6 months in the HDF group (*p* = 0.006). The difference in the rate of change between HD and HDF was 0.097 nmol/L per 6 months (*p* < 0.001). Between six and twelve months, the rate of change in the HD group was 0.039 nmol/L per 6 months (*p* = 0.06), and −0.028 nmol/L per 6 months in the HDF group (*p* = 0.19). The difference in the rate of change between the HD and the HDF group was 0.067 nmol/L per 6 months (*p* = 0.02). Adjustment for change in eGFR did not change the effect of HDF (Table 5). 

In HDF patients, the tertiles of median delivered convection volume during the first year were ≤17.2 L per session, 17.2 to 20.1 L per session and ≥20.1 L per session. There was no association between the decrease in CTGF between baseline and six months and the amount of convection volume (Table 6).

## 3. Discussion

In the present study, we show that plasma CTGF is independently related to RKF, previous CVD, IL-6 and B2M in maintenance HD patients, which are all important predictors of mortality in this population [20,21,22,23]. In addition, CTGF levels depend on the underlying renal disease and are higher in PKD, TIN, and renal vascular disease. Second, all-cause mortality risk is increased in HD patients with high baseline plasma CTGF, whereas no relation was found between CTGF and risk of cardiovascular events, cardiovascular mortality, and infectious mortality. Third, treatment with HDF reduced plasma CTGF concentration over time, while CTGF concentration increased in patients treated with conventional low-flux HD.

### 3.1. Determinants of Plasma CTGF Concentrations 

CTGF levels in our patient group were approximately 10- to 20-fold higher than in healthy subjects [19,24,25] and were inversely related to eGFR, even at very low levels (2.6 (1.2–5.1) mL/min/1.73 m^2^). This is compatible with the notion that N-CTGF, which is the predominant form of CTGF detected in plasma, is almost exclusively removed by the kidneys [6,7]. We found a positive relation between CTGF and dialysis vintage. This is in line with previous observations that retained middle molecules accumulate gradually over time in chronic dialysis patients [20].

CTGF was positively related to previous CVD, in particular to previous PVD. As CTGF expression is strongly upregulated in atherosclerotic plaques [14] and symptomatic PVD is an indication of widespread atherosclerotic disease throughout the body, elevated CTGF concentrations might reflect increased production by atherosclerotic lesions. Whether high plasma CTGF concentrations in HD patients are merely a reflection of the overall burden of CVD, or play a causative role in atherogenesis remains to be determined. In support of the latter, it was shown that a CTGF promoter polymorphism that causes increased CTGF expression was associated with increased risk of cardiovascular morbidity and mortality in HD patients [26]. In addition, carotid atherosclerotic lesions in mice treated for three weeks with CTGF-neutralizing antibodies, showed reduced macrophage deposition, CTGF expression, and plaque volume [27].

We found an independent positive relationship between CTGF and both IL6 and B2M, which are both markers of inflammation and predictors of mortality in dialysis patients [20,23]. The underlying mechanisms are unclear, but a study in mice showed that intravenous injection of recombinant human CTGF peptide induced renal inflammatory responses, with locally increased IL-6 levels [28]. Hence, CTGF might be upregulated by inflammatory processes or vice versa. The relationship between CTGF and B2M is less clear, but might also be related to inflammation, since B2M has been shown to decrease when inflammation is reduced after introduction of ultrapure dialysis fluids in HD patients [29,30].

Interestingly, CTGF was higher in patients with PKD than in other underlying renal diseases. Data on a possible relationship between CTGF and PKD are scarce. CTGF is increased in mice models of PKD [31,32], downstream of the nuclear expression of the Yes-associated protein (YAP), which is upregulated in cystic epithelia in mice and humans with advanced PKD and presumed to be involved in cyst growth [32]. Mutations causing nephronopthisis, a recessive cystic kidney disease in children, have been shown to increase CTGF levels [33]. CTGF might also be increased downstream of transforming growth factor β signalling, which is increased during cyst expansion and fibrosis in more advanced stages of PKD in *Pkd1-*mutant mouse models and in humans with autosomal dominant PKD (ADPKD) [34]. Tubulointerstitial fibrosis has been identified as an important manifestation in ADPKD, associated with an increased rate of progression to ESKD [35]. The detection of CTGF expression in cells adjacent to cystic epithelium and in fibrotic renal tissue in an ADPKD-rat model, suggests a role in the local response to cyst development and tubulointerstitial fibrosis [36]. Future research should clarify whether CTGF is involved in this. In patients with TIN, plasma CTGF concentrations were also high, which is consistent with the previously observed strong correlation between the extent of tubulointerstitial damage and the number of CTGF mRNA positive cells per surface area in 65 human renal biopsy specimens of various renal diseases [37].

While CTGF has been implicated in the pathogenesis of diabetic complications, we did not observe a difference in CTGF concentrations between patients with or without DM. This suggests that a direct effect of DM on plasma CTGF levels in HD patients may be overruled by other factors such as the presence of advanced vascular and kidney disease.

A limitation of the study is that the diagnoses of the underlying renal diseases were made by a number of different attending nephrologists, and were often not biopsy proven. However, possible diagnostic variability is compensated at least in part by the size of our study population. The diagnoses of PKD and diabetic kidney disease were relatively clear-cut. The strength of our study is the large, well described group of HD patients that was studied.

In conclusion, in chronic HD patients, CTGF concentrations depend on RKF and underlying renal disease and relate positively to previous CVD and inflammation. The role of CTGF in the pathogenesis of certain renal diseases, in particular PKD, deserves further exploration in clinical and preclinical studies.

### 3.2. CTGF and All-cause Mortality

Previously, the association between CTGF and all-cause mortality was studied in 386 patients with type 1 DM and was shown to be related to all-cause mortality in 198 patients with DM. One standard deviation increase in CTGF (from 0.38 to 0.72 nmol/L) was associated with a 40% increase in mortality risk [19]. In the present study, we also observed an association between baseline plasma CTGF and all-cause mortality, although the magnitude of the relationship was much smaller (9% increase in mortality risk per 1 nmol/L increase in plasma CTGF). Mortality risk was substantially increased by 71% in the highest CTGF quartile (CTGF > 4.3 nmol/L), as compared to the lowest (CTGF < 2.7 nmol/L). 

A relationship between CTGF and cardiovascular outcomes during follow-up was not observed. This is in contrast with the finding that there is a positive association between CTGF concentrations and previous CVD and previous evidence of a pathogenic role of CTGF in cardiovascular fibrosis [3,9,13] Possibly, the harmful effect of CTGF is overruled by other factors such as derangement of calcium-phosphate metabolism and presence of advanced cardiovascular fibrosis at the start of the study. We neither found a relationship with infectious mortality. However, CVD or infection as a cause of mortality occurred in only 32% and 25% of all-cause mortality, respectively. The absence of a clear association with cardiovascular outcome or infection suggests that CTGF may not reflect one specific disease process. This might be explained by considering fibrosis as a common final pathway of chronic disease processes of diverse etiology, including inflammation, ischemia and metabolic derangement, and CTGF as a key determinant of activity of fibrotic processes, reflecting the overall fibrosis burden. 

### 3.3. CTGF Change over Time, HDF versus HD 

Plasma CTGF increased in patients on HD and was lowered by HDF over time, which is in agreement with our previous report showing that CTGF is not removed by low flux HD and is reduced by a single HDF session [6]. While CTGF was lowered by 68% during a single session, one year of treatment resulted in a decrease in pre-dialysis concentration of approximately 15%. Presumably, this difference is explained by post-dialysis redistribution from extravascular compartments, which has been described for other middle molecules such as B2M [38], and by interdialytic accumulation of de novo produced CTGF due to absent or minimal clearance.

Clearance of middle molecules is enhanced by convection [39]. While CTGF decreased in patients treated with HDF and increased in patients with HD, we did not observe a relationship between decline in CTGF and the amount of convection volume that was delivered in patients on HDF. This might indicate that CTGF diffusion from extra-vascular compartments to the blood is rate limiting, as was described for B2M [40]. Other possible explanations are that the reduction of pre-dialysis CTGF by convection is overruled by much more substantial post-dialysis redistribution of CTGF or that the maximum decrease in CTGF is reached with ≤17 L of convection volume. 

A limitation is that blood samples were not collected at time points between baseline and six months and after 12 months. Therefore, it was not possible to determine at what time point the lower CTGF concentrations in HDF patients were reached, although the analysis suggested that the greatest decline in CTGF occurred during the first six months of therapy (Figure 1, Table 6), as is the case for B2M [39]. Furthermore, we did not study CTGF at time points beyond one year, but the resemblance with B2M suggests a sustained effect. The second limitation is that dialysate collection was not performed. Therefore, we were not able to determine whether the reduction of plasma CTGF in HDF patients was caused by decreased production, increased clearance, or adhesion to the dialyzer. However, the effect of a single HDF session suggests either increased clearance or adhesion. The strengths of our study are the large group of dialysis patients, the large number of events and the randomized study design.

It is unknown whether circulating CTGF, and in particular its N-terminal fragment (CTGF-N), the predominant form of CTGF in plasma, exerts systemic toxicity. An in vitro study reported that the N-terminal fragment is biologically active by mediating myofibroblast differentiation and collagen synthesis [41], but in vivo evidence of profibrotic activity is lacking. Future studies should establish whether plasma CTGF-N has adverse biological effects or merely is an inactive proteolytic product shed from the extracellular matrix into the circulation. 

In conclusion, in HD patients, high plasma CTGF is related to an increased risk of all-cause mortality, but not to risk of cardiovascular morbidity and mortality. Treatment with HDF reduces plasma CTGF, while in patients treated with conventional low-flux HD CTGF concentration increases over time. It remains to be determined whether lowering circulating CTGF is beneficial in ESKD patients.

## 4. Methods

### 4.1. General Methods 

In the present study, data from 404 HD patients from 17 dialysis centres in The Netherlands and one centre in Norway in which storage of additional blood samples for non-routine laboratory assessments was logistically feasible, who were enrolled in the CONvective TRAnsport STudy (CONTRAST) between June 2004 and January 2010, were used. CONTRAST is a randomized controlled trial (ISRCTN38365125) that compares the effects of low-flux HD and online HDF on all-cause mortality and cardiovascular morbidity and mortality, as described [42]. The study was approved by the Medical Research Ethics Committee, VU Medical Center, Amsterdam, the Netherlands, on 3 December 2003 (ClinicalTrials gov identifier: NCT00205556). Patients were eligible for inclusion if they were treated with low-flux HD two or three times per week for at least two months, with a minimum dialysis single pool Kt/V urea (spKt/V_urea_) of 1.2. Exclusion criteria were aged below 18 years, treatment with HDF or high-flux HD in the six months preceding randomization, severe non-adherence to the dialysis prescription in terms of frequency and/or duration of dialysis treatment, a life expectancy less than three months due to causes other than kidney disease, and participation in another clinical intervention study evaluating cardiovascular outcome. Randomization was performed centrally by a computer-based randomization service (Julius Center University Medical Center, Utrecht, the Netherlands) into a 1:1 ratio and stratified per participating centre. The study was conducted in accordance with the Declaration of Helsinki and approved by the medical ethics review boards of all participating centres. Written informed consent was obtained from all patients prior to randomization. With this study, three different research questions were addressed: 1) What are the determinants of plasma CTGF in HD patients? 2) Is CTGF related to all-cause mortality and cardiovascular morbidity and mortality in HD patients? 3) Can pre-dialysis CTGF concentrations be lowered over time by HDF as compared to low-flux HD?

### 4.2. Dialysis Procedures

Treatment times were fixed during follow-up in both treatment arms, unless spKt/V_urea_ was below 1.2. Online HDF was performed in the post-dilution mode with a target convection volume of 6 L/h. Blood flow rates could be increased in the HDF arm to improve convection volumes. For HDF, synthetic high-flux dialyzers were used (FX80: 35% and FX100: 9% [Fresenius Medical Care, Bad Homburg, Germany], Polyflux 170H: 34% and Polyflux 210H: 21% [Gambro Corporation AB, Lund, Sweden], or other dialyzers: 1%, at 3 months). HD patients were treated with synthetic low-flux dialyzers (F8HPS: 56% and F10HPS: 2% [Fresenius], Polyflux 14 L: 2%, Polyflux 17 L: 30% and Polyflux 21 L: 3% [Gambro], or other: 6%, at three months). All patients were treated with ultrapure dialysis fluids, defined as less than 0.1 colony forming units per mL and less than 0.03 endotoxin units per mL [43]. When hemodiafiltration could temporarily not be performed e.g. due to technical problems with the water installation, or holiday bookings, patients concerned were treated with high-flux membranes. Routine patient care was performed according to national and international quality of care guidelines.

### 4.3. Data Collection

At baseline, standardized forms were used to collect demographical, clinical and laboratory data, which included age, sex, body mass index (BMI, kg/m^2^), pre-dialysis systolic and diastolic blood pressure (mmHg), medical history (including the cause of kidney failure, presence of diabetes mellitus (DM) and previous CVD), medication, estimated glomerular filtration rate (eGFR, mL/min/1.73m^2^) and residual kidney function (RKF, yes/no). Furthermore, dialysis related data were collected as described previously, including duration of dialysis (dialysis vintage), vascular access type, number of treatments per week, blood flow rate, and dialysis adequacy expressed as spKt/V calculated with the second-generation Daugirdas formula [44]. 

Previous CVD was defined as a history of angina pectoris, myocardial infarction, prior coronary revascularization (percutaneous transluminal coronary angioplasty and coronary artery bypass surgery), stroke, transient ischemic attack and/or peripheral vascular disease (PVD) (intermittent claudication, amputation, percutaneous transluminal angioplasty and peripheral bypass surgery). Dialysis vintage was determined as the sum of time spent on HD or peritoneal dialysis before inclusion in CONTRAST.

At each three-monthly visit, data on clinical events, dialysis treatment, medication, and laboratory values were recorded, which included data on blood flow rate, treatment time, infusion volume, intradialytic weight loss, and pre-dialysis blood pressure. In HDF patients, infusion volumes (liters per treatment) were reported as the mean value of three consecutive treatment sessions preceding the quarterly visit. Convection volumes (liters per treatment) were calculated as the sum of the intradialytic weight loss and the substitution volume per session. For calculation of the delivered convection volume the number of hemodiafiltration treatments in the past three months was assessed. The mean delivered convection volume during the first year of the trial was estimated with the following formula: Mean delivered convection volume = (hemodiafiltration treatments/ total number of treatments) × mean convection volume of the three treatments preceding the quarterly visit.

At each visit, blood samples were drawn prior to dialysis, and if appropriate, after the session. Blood samples for non-routine measurements were taken at baseline, six, and twelve months before the dialysis session, placed immediately on ice, and centrifuged within 30 min, at 1500 g for 10 min and stored at –80°C until assayed.

In patients with a urinary production of more than 100 mL per day, interdialytic 24-h urinary samples were collected. Estimated glomerular filtration rate (eGFR) was calculated as the mean of creatinine and urea clearance and adjusted for body surface area (mL/min/1.73m^2^) [45]. The plasma concentrations used for this calculation were the mean of the values before and after dialysis. Patients with a urinary production below 100 mL per day were considered anuric and eGFR was reported as zero and RKF as ‘no’. The second generation Daugirdas formula was used to calculate spKt/V_urea_ [44]. 

### 4.4. Laboratory Measurements 

CTGF plasma concentrations were determined by sandwich ELISA, using two specific antibodies (FibroGen Inc, San Francisco, CA, USA) directed against two distinct epitopes in the amino-terminal fragment of CTGF, detecting both full length CTGF and the N-fragment, as described [7]. Microtiter plates (Maxisorb; Nunc, Roskilde, Denmark) were coated with capture anti-CTGF monoclonal antibody (5 μg/mL; FibroGen). Subsequently, ten-fold diluted plasma samples and standards (recombinant human CTGF; FibroGen, San Francisco, CA) were added and incubated with detection antibody conjugated directly to alkaline phosphatase (0.5 μg/mL; FibroGen). Para-nitrophenylphosphate (Sigma, St. Louis, MO, USA) was used as a substrate for the colorimetric reaction. Intra- and inter-assay coefficients of variation were within 10%. To improve inter-assay precision, control/ reference plasma samples were used on each plate to calculate a correction factor. Matrix correction was applied. Assay sensitivity (lower limit of detection) was 20 pmol/L.

High sensitivity C reactive protein (CRP) was measured centrally with a particle-enhanced immunoturbidimetric assay on a Roche-Hitachi analyzer (Roche Diagnostics GmbH, Mannheim, Germany), with a lower quantification limit of 0.1 mg/L. The intra-assay variation was between 1.9% at a concentration of 0–1.0 mg/L and 0.3% at a concentration of 2.0–4.0 mg/L, the inter-assay variation was 1.9% at a concentration of 0.67 mg/L and 1.2% at a concentration of 3.64 mg/L.

Interleukin-6 (IL-6) (pg/mL) was measured centrally with an immunometric assay (colorimetric) (Sanquin, Amsterdam, The Netherlands), with a lower quantification limit of 0.35 pg/mL. The intra-assay variation was 12% at a concentration of 1 pg/mL and 8% at a concentration of 3 pg/mL, the inter-assay variation was 19% at a concentration of 0.35 pg/mL (which is the lower limit of quantification) and 12% at a concentration of 2.3 pg/mL. Routine laboratory tests were performed in the different participating centres using standard techniques.

### 4.5. Data Analysis

#### 4.5.1. Determinants of Plasma CTGF 

To identify determinants of plasma CTGF at baseline, a backward multivariable regression model was applied, which included several demographic, clinical, and laboratory factors. Due to the skewed distribution, the natural logarithm of CTGF, CRP and IL-6 was used. CTGF was used as the dependent variable in the regression models. A *p*-value of 0.10 was used to exclude variables from the model. Variables were chosen, because they are established cardiovascular risk factors, or because we expected a relationship with CTGF based on pathophysiological considerations. The initial backward model started off with age, sex, history of CVD, smoking, pulse pressure, presence of DM, underlying renal disease, BMI, eGFR, ultrafiltration (UF) requirement, dialysis vintage, albumin, CRP, IL-6, beta-2-microglobulin (B2M), and use of renin angiotensin aldosterone system (RAAS) blockers, beta blockers, calcium antagonists and statins. Since ‘history of CVD’ was a composite variable, we additionally explored whether CTGF was related to its individual components, i.e. coronary heart disease (CHD), cerebrovascular accidents (CVA), PVD. Unstandardized residuals of the model were checked on normality. All analyses were performed with SPSS (version 18; SPSS Inc. Headquarters, Chicago, IL, USA).

#### 4.5.2. CTGF and All-cause Mortality, Cardiovascular Events, Cardiovascular Mortality and Infectious Mortality 

The primary study outcome was all-cause mortality. Follow-up of patients with respect to mortality was complete. Participants in the trial continued to be followed even after they received a kidney transplant, switched to peritoneal dialysis, moved to another non-CONTRAST centre or discontinued participation for other reasons.

The secondary endpoint was a composite of fatal and non-fatal cardiovascular events. Cardiovascular events were defined as death from cardiovascular causes, non-fatal myocardial infarction or stroke, therapeutic coronary procedure (coronary artery bypass graft, percutaneous transluminal coronary angioplasty and/or stenting), therapeutic carotid procedure (endarterectomy and/or stenting), vascular intervention (revascularisation, percutaneous transluminal angioplasty and/or stenting), or amputation. 

Another secondary endpoint was cause specific death, i.e. cardiovascular and infectious death. An independent Endpoint Adjudication Committee, whose members were not aware of the treatment assignments, reviewed source documentation for all primary outcome events (deaths), as well as non-fatal cardiovascular events. 

Cox proportional hazards regression models were used to study the relation between CTGF at baseline and mortality. Thereafter, adjustment was performed for potential confounding variables, i.e. those factors that after addition to the model changed the magnitude of the relationship between CTGF and mortality by more than 5%. Potential confounders thus identified were presence of CVD, presence of polycystic kidney disease (PKD) as underlying renal disease, eGFR, use of renin angiotensin aldosterone system blockers (RAAS blockers), age, sex, presence of DM, CRP, and albumin. Since CRP and albumin might be intermediates in the relation between CTGF and mortality, a model without adjustment for these variables was also performed. In addition, an ‘on-treatment’ analysis was performed, where patients were censored 28 days after they left the study. To explore a dose response relationship between CTGF and mortality, CTGF concentrations of the complete cohort were divided into quartiles, with the lowest quartile serving as the reference group. Finally, using similar models as for all-cause mortality, the relation between CTGF concentrations and different outcomes was explored, i.e., fatal or non-fatal cardiovascular events, and death from CVD and death from infection. 

#### 4.5.3. CTGF Change over Time, HDF versus HD 

The main outcome was the difference in rate of change in CTGF concentration (percentage per 6 months) between HDF and HD patients during one year of treatment. 

A linear mixed effects model with a random intercept and random slope was applied to compare the rate of change in CTGF over time between both treatment arms. The natural logarithm of CTGF (Ln-CTGF) was used for the analyses to improve the fit of the model. It was explored whether the effect of HDF on Ln-CTGF changed over time using a linear mixed effect model. Since a statistically significant interaction was found between HDF treatment and time, data on the rate of change over time are presented in two time periods: Baseline to six months and six to twelve months. To explore the influence of changing eGFR over time on the change in CTGF, an additional linear mixed model analysis with adjustments for eGFR was performed.

Finally, it was explored whether the change in CTGF between baseline and the six-month visit depended on delivered convection volume in HDF patients. For this purpose, the actual (on-treatment) delivered convection volume was divided into tertiles, which were introduced in a multivariable regression model as dummy variables. Patients who were treated by low-flux HD served as reference group (no convection volume). Adjustment for potential confounding factors, i.e. variables that were related to change in CTGF between baseline and the six-month visit, was performed. Unstandardized residuals of the regression model were checked on normality. 

The linear mixed effects model was performed with R (version 2.9.2; 2009 The R Foundation for Statistical Computing). The other analyses were performed with SPSS software (version 18.0; SPSS Inc. Headquarters, Chicago, IL, USA).

## Figures and Tables

**Table 1:** Characteristics of participants at baseline.

Characteristic	On-Line HDF (*n* = 199)	Low Flux HD (*n* = 205)
Age (years)	64 ± 14.3	63 ± 13.6
Male sex—n (%)	121 (61)	130 (63)
Region		
Netherlands—n (%)	193 (96)	197 (96)
Norway—n (%)	7 (4)	8 (4)
History of cardiovascular disease—n (%)	90 (45)	87 (42)
Diabetes mellitus—n (%)	46 (23)	39 (19)
Dialysis vintage (years)	1.7 (0.9 to 3.1)	2.0 (0.9 to 3.7)
Underlying renal disease—n (%)		
Glomerulonephritis	10 (5%)	11 (6%)
Tubulointerstitial nephritis	17 (9%)	14 (7%)
Polycystic kidney disease	10 (5%)	10 (5%)
Other congenital/hereditary diseases	2 (1%)	0
Renal vascular disease	37(19%)	47 (24%)
Diabetic nephropathy	22 (11%)	22 (11%)
Multisystem disease	8 (4%)	7 (4%)
Other	19 (10%)	22 (11%)
Unknown	74 (37%)	72 (36%)
Systolic blood pressure (mmHg) ^	147 ± 22	149 ± 21
Diastolic blood pressure (mmHg) ^	75 ± 12	76 ± 12
Pulse pressure, pre-dialysis (mmHg)	69 ± 18	68 ± 16
Body mass index, after dialysis (kg/m^2^)	24.7 ± 4.9	25.3 ± 4.7
Vascular access– n (%)		
Arteriovenous fistula	162 (81)	176 (86)
Graft	31 (16)	22 (11)
Central catheter	5 (3)	5 (2)
Duration of a dialysis session (hours)	4.0 (3.5 to 4.0)	4.0 (3.5 to 4.0)
Number of treatments/week	2.90 ± 0.30	2.95 ± 0.23
Blood flow (mL/min)	300 (300 to 325)	300 (299 to 326)
Dialysis single pool Kt/V_urea_	1.35 (1.23 to 1.52)	1.33 (1.23 to 1.47)
Residual kidney function—n (%) *	119 (60)	111 (54)
Estimated glomerular filtration rate (mL/min/1.73m^2^) ^	2.8 (1.3 to 5.1)	2.5 (1.1 to 5.1)
Hemoglobin (mmol/L) ^	7.4 ± 0.8	7.3±0.7
Phosphorus (mmol/L) ^	1.68 ± 0.56	1.65 ± 0.47
Beta-2-microglobulin (mg/L) ^	28.5 ± 11.5	31.7 ± 11.9
Albumin (g/L) ^	35.8 ± 4.8	36.0 ± 4.8
Creatinine (μmol/L) ^	837 ± 243	896 ± 255
Connective Tissue Growth Factor (nmol/L) ^	3.5 (2.7 to 4.3)	3.5 (2.8 to 4.4)
Ln-CTGF (nmol/L) ^	1.24 ± 0.39	1.26 ± 0.39
Hs-CRP (mg/L) ^	4.06 (1.43 to 10.43)	3.88 (1.31 to 10.28)
IL-6 (pg/mL) ^	2.11 (1.26 to 4.08)	1.98 (1.18 to 3.42)
Prescribed medication—n (%)		
RAAS blocker	108 (54)	107 (52)
Beta blocker	101 (51)	126 (61)
Calcium antagonist	63 (32)	72 (35)
Statin	104 (52)	97 (47)

Data are presented as mean ± SD, medians (interquartile range) or absolute number (percentage). HDF, online hemodiafiltration; HD, low-flux hemodialysis; AV-fistula, arteriovenous fistula; hsCRP, high sensitivity C- reactive protein; IL-6, Interleukin 6; Ln-CTGF, natural logarithm of CTGF. To convert hemoglobin in mmol/L to g/dL divide by 0.62; albumin in g/L to g/dL, divide by 10; creatinine in μmol/L to mg/dL divide by 88.4 ^ Pre-dialysis * Residual kidney function if diuresis > 100 mL/24 h.

**Table 2:** Multivariable analysis of determinants of plasma Ln-CTGF in HD patients.

Determinants	β	*p*	Change in CTGF (%) (95% CI) *
Previous CVD	0.099	0.006	10.4 (2.8 to 18.5)
Dialysis vintage	0.031	<0.001	3.1 (1.7 to 4.5)
Pulse pressure	0.002	0.059	0.2 (0 to 0.4)
Ln-IL-6	0.075	<0.001	7.8 (4.1 to 11.7)
β2-microglobulin	0.003	0.045	0.3 (0 to 0.7)
eGFR	–0.033	<0.001	−3.3 (−4.4 to −2.1)
RAAS-blocker use	–0.068	0.052	−6.6 (−12.8 to 0.1)
UF	0.034	0.091	3.5 (−0.6 to 7.7)
Underlying renal disease			
Glomerulonephritis	0.050	0.484	5.1 (−8.7 to 20.8)
Tubulointerstitial nephritis	0.222	0.002	24.9 (8.5 to 43.6)
Polycystic kidney disease	0.403	<0.001	49.6 (28.1 to 74.5)
Renal vascular disease	0.115	0.041	12.2 (5.0 to 25.4)
Diabetic nephropathy	0.103	0.120	10.8 (−23.7 to 26.1)
Multisystem disease	0.078	0.387	8.1 (−9.4 to 29.2)
Other	0.030	0.682	3.0 (−10.8 to 18.9)
Unknown	reference		

CTGF, connective tissue growth factor; HD, low-flux hemodialysis; CVD, cardiovascular disease; Ln, natural logarithm; Ln-IL-6, natural logarithm of interleukin 6; eGFR, estimated glomerular filtration rate; RAAS-blocker, renin angiotensin aldosterone system blocker; UF, ultrafiltration as calculated by the mean difference between pre- and post-dialysis weight on 3 different occasions; CI, confidence. *R*^2^ = 0.37. * Relative increase in CTGF if β is calculated back from the natural logarithm. If the determinant increases by 1 unit or changes from 0 to 1 for a dichotomous variable, Ln-CTGF will increase with β and CTGF will be multiplied by e^β^.

**Table 3:** Hazard ratios for mortality and cardiovascular events for each increase of plasma CTGF with 1 nmol/L.

Outcome	Model	*N*	#Events	Hazard Ratio (95% CI)	*P*
All-cause mortality	I II III	404 384 382	158 151 149	1.10 (1.01–1.19) 1.09 (1.00–1.19) 1.09 (0.99–1.19)	0.02 0.05 0.07
Fatal and non-fatal cardiovascular events	I IV	404 362	131 121	1.06 (0.96–1.16) 1.03 (0.94–1.14)	0.24 0.51
Cardiovascular mortality	I V	404 394	50 46	0.99 (0.84–1.18) 1.01 (0.85–1.19)	0.93 0.96
Infectious mortality	I VI	404 391	40 39	1.14 (0.99–1.31) 1.06 (0.92–1.23)	0.08 0.42

Model I: univariate. Model II: adjustment for age, sex, presence of cardiovascular disease (CVD), presence of diabetes mellitus, estimated glomerular filtration rate (eGFR), polycystic kidney disease (PKD), use of RAAS-blockers. Model III: model II with additional adjustment for CRP and albumin. Model IV: model III with additional adjustment for dialysis vintage and serum β2-microglobulin. Model V: adjustment for age CVD, eGFR, PKD, albumin. Model VI: adjustment for age, CVD, DM, serum albumin. *N* = number of patients at risk at baseline. **#** Fatal and non-fatal cardiovascular events are defined as death from cardiovascular causes, non-fatal myocardial infarction or stroke, therapeutic coronary procedure (coronary artery bypass graft, percutaneous transluminal coronary angioplasty and/or stenting), therapeutic carotid procedure (endarterectomy and/or stenting), vascular intervention (revascularisation, percutaneous transluminal angioplasty and/or stenting), or amputation. Infectious mortality is defined as death from infectious causes.

**Table 4:** Hazard ratios of plasma CTGF quartiles for mortality and cardiovascular events.

Outcome	Model	Hazard Ratio (95% CI)			
		Quartile 1	Quartile 2	Quartile 3	Quartile 4
All-cause mortality	I II	1.00 (reference) 1.00 (reference)	1.17 (0.73–1.87) 1.15 (0.69–1.91)	1.23 (0.78–1.96) 1.09 (0.63–1.88)	1.60 (1.03–2.50) 1.71 (1.02–2.88)
Fatal and non-fatal cardiovascular events	I III	1.00 (reference) 1.00 (reference)	1.35 (0.80–2.26) 1.42 (0.81–2.50)	1.85 (1.12–3.04) 1.91 (1.08–3.38)	1.41 (0.83–2.38) 1.63 (0.86–3.08)

Model I: univariate. Model II: adjustment for age, sex, presence of cardiovascular disease, presence of diabetes mellitus, estimated glomerular filtration rate, polycystic kidney disease, use of RAAS-blockers, CRP and albumin. Model III: model II with additional adjustment for dialysis vintage and serum β2-microglobulin. Plasma CTGF concentrations: quartile 1: <2.7 nmol/L, quartile 2: 2.7–3.5 nmol/L, quartile 3: 3.5–4.3 nmol/L, quartile 4: >4.3 nmol/L.

**Table 5:** Plasma CTGF over time in patients treated with hemodialysis and hemodiafiltration.

Ln-CTGF	Model	Change in Plasma CTGF Concentration (Δ) per 6 Months					
		HD (N = 205)		HDF (N = 199)		HDF versus HD	
		Δ	*p*	Δ	*p*	Δ	*p*
**0–6 Months**							
Ln-CTGF (nmol/L) (mean (95% CI))	I II	0.047 (0.013 to 0.081) 0.031 (−0.0055 to 0.062)	0.007 0.10	−0.050 (−0.086 to −0.015) −0.058 (−0.096 to −0.019)	0.006 0.003	−0.097 (−0.147 to −0.048) −0.088 (−0.133 to −0.036)	<0.001 0.001
CTGF (%) *	I II	+4.9 +3.1		−4.8 −5.4		−8.9 −8.1	
**6–12 Months**							
Ln-CTGF (nmol/L) (mean (95% CI))	I II	0.039 (−0.002 to 0.080) 0.042 (−0.001 to 0.085)	0.06 0.06	−0.028 (−0.070 to 0.014) −0.034 (−0.077 to 0.011)	0.19 0.13	−0.067 (−0.123 to -0.009) −0.075 (−0.136 to -0.014)	0.02 0.02
CTGF (%) *	I II	+4.1 +4.3		−2.7 −3.2		−6.3 −7.0	

Model I: univariate. Model II: adjustment for eGFR over time. HD, low-flux hemodialysis; HDF, online hemodiafiltration; Ln-CTGF, natural logarithm of CTGF. * Calculated by e^ΔLn-CTGF.^

**Table 6:** Effect of HDF versus HD on the change in plasma CTGF between baseline and six months in strata of convection volume.

	Mean Δ Ln-CTGF * (nmol/L/6 Months) (95% CI)	*p*
Convection Tertile 1	−0.038 (−0.070 to −0.003)	0.03
CTGF (%) **	−3.6	-
Convection Tertile 2	−0.044 (−0.075 to -0.011)	0.009
CTGF (%) **	−4.2	-
Convection Tertile 3	−0.046 (−0.075 to −0.015)	0.004
CTGF (%) **	−4.4	-

Adjustment for age, CVD, dialysis vintage, dialysis frequency, eGFR, use of RAAS blockers, CTGF at baseline. HDF, online hemodiafiltration; HD, low-flux hemodialysis; Ln-CTGF, natural logarithm of CTGF. Tertile 1: ≤17.2 L per session, Tertile 2: 17.2–20.1 L per session, Tertile 3: ≥20.1 L per session * Mean Δ Ln-CTGF = mean Δ Ln-CTGF_HDF_ – mean Δ Ln-CTGF_HD_ ** Calculated by e^ΔLn-CTGF.^

## Local Investigators

**The Netherlands:** Academic Medical Center, Amsterdam – MG Koopman; Dianet Dialysis Centers, Utrecht – M Kooistra and B van Jaarsveld; Gelderse Vallei Hospital, Ede – GW Feith; Haga Hospital, The Hague – M. van Buren; Jeroen Bosch Hospital, ’s Hertogenbosch – EK Hoogeveen; Maasstad Hospital, Rotterdam – PJ van de Ven; Martini Hospital, Groningen – TK Kremer Hovinga; Medical Center Alkmaar, Alkmaar – WA Bax; Onze Lieve Vrouwe Gasthuis, Amsterdam – JO Groeneveld; Rijnstate Hospital, Arnhem – LJM Reichert; Slingeland Hospital, Doetinchem – J Huussen; University Medical Center St Radboud, Nijmegen –HW van Hamersvelt; University Medical Center Utrecht, Utrecht – WH Boer, EL Penne, NC van der Weerd and AH Mazairac; VieCuri Medical Center, Venlo – WH van Kuijk; VU Medical Center, Amsterdam – MG Vervloet, EL Penne, NC van der Weerd and PM ter Wee; Zeeuws-Vlaanderen Hospital, Terneuzen – IMPMJ Wauters. **Norway:** Haukeland University Hospital, Bergen – I Sekse.

## References

[1] Ramazani Y., Knops N., Elmonem M.A., Nguyen T.Q., Arcolino F.O., van den Heuvel L., Levtchenko E., Kuypers D., Goldschmeding R. (2018). Connective tissue growth factor (CTGF) from basics to clinics. Matrix Biol. J. Int. Soc. Matrix Biol..

[2] Dendooven A., Gerritsen K.G., Nguyen T.Q., Kok R.J., Goldschmeding R. (2011). Connective tissue growth factor (CTGF/CCN2) ELISA: A novel tool for monitoring fibrosis. Biomark. Biochem. Indic. Expo. Response Susceptibility Chem..

[3] Daniels A., van Bilsen M., Goldschmeding R., van der Vusse G.J., van Nieuwenhoven F.A. (2009). Connective tissue growth factor and cardiac fibrosis. Acta Physiol. (Oxf. Engl.).

[4] Ruperez M., Lorenzo O., Blanco-Colio L.M., Esteban V., Egido J., Ruiz-Ortega M. (2003). Connective tissue growth factor is a mediator of angiotensin II-induced fibrosis. Circulation.

[5] Ruster C., Wolf G. (2011). Angiotensin II as a morphogenic cytokine stimulating renal fibrogenesis. J. Am. Soc. Nephrol. JASN.

[6] Gerritsen K.G., Abrahams A.C., Peters H.P., Nguyen T.Q., Koeners M.P., den Hoedt C.H., Dendooven A., van den Dorpel M.A., Blankestijn P.J., Wetzels J.F. (2012). Effect of GFR on plasma N-terminal connective tissue growth factor (CTGF) concentrations. Am. J. Kidney Dis. Off. J. Natl. Kidney Found..

[7] Gerritsen K.G., Peters H.P., Nguyen T.Q., Koeners M.P., Wetzels J.F., Joles J.A., Christensen E.I., Verroust P.J., Li D., Oliver N. (2010). Renal proximal tubular dysfunction is a major determinant of urinary connective tissue growth factor excretion. Am. J. Physiol. Ren. Physiol..

[8] Ohnishi H., Oka T., Kusachi S., Nakanishi T., Takeda K., Nakahama M., Doi M., Murakami T., Ninomiya Y., Takigawa M. (1998). Increased expression of connective tissue growth factor in the infarct zone of experimentally induced myocardial infarction in rats. J. Mol. Cell. Cardiol..

[9] Koitabashi N., Arai M., Kogure S., Niwano K., Watanabe A., Aoki Y., Maeno T., Nishida T., Kubota S., Takigawa M. (2007). Increased connective tissue growth factor relative to brain natriuretic peptide as a determinant of myocardial fibrosis. Hypertension (Dallas Tex. 1979).

[10] Koitabashi N., Arai M., Niwano K., Watanabe A., Endoh M., Suguta M., Yokoyama T., Tada H., Toyama T., Adachi H. (2008). Plasma connective tissue growth factor is a novel potential biomarker of cardiac dysfunction in patients with chronic heart failure. Eur. J. Heart Fail..

[11] Rickard A.J., Morgan J., Chrissobolis S., Miller A.A., Sobey C.G., Young M.J. (2014). Endothelial cell mineralocorticoid receptors regulate deoxycorticosterone/salt-mediated cardiac remodeling and vascular reactivity but not blood pressure. Hypertension (Dallas Tex. 1979).

[12] Ahmed M.S., Oie E., Vinge L.E., Yndestad A., Oystein Andersen G., Andersson Y., Attramadal T., Attramadal H. (2004). Connective tissue growth factor—A novel mediator of angiotensin II-stimulated cardiac fibroblast activation in heart failure in rats. J. Mol. Cell. Cardiol..

[13] Dorn L.E., Petrosino J.M., Wright P., Accornero F. (2018). CTGF/CCN2 is an autocrine regulator of cardiac fibrosis. J. Mol. Cell. Cardiol..

[14] Cicha I., Yilmaz A., Klein M., Raithel D., Brigstock D.R., Daniel W.G., Goppelt-Struebe M., Garlichs C.D. (2005). Connective tissue growth factor is overexpressed in complicated atherosclerotic plaques and induces mononuclear cell chemotaxis in vitro. Arterioscler. Thromb. Vasc. Biol..

[15] Leeuwis J.W., Nguyen T.Q., Theunissen M.G., Peeters W., Goldschmeding R., Pasterkamp G., Vink A. (2010). Connective tissue growth factor is associated with a stable atherosclerotic plaque phenotype and is involved in plaque stabilization after stroke. Stroke.

[16] Ponticos M. (2013). Connective tissue growth factor (CCN2) in blood vessels. Vasc. Pharmacol..

[17] Gerritsen K.G., Falke L.L., van Vuuren S.H., Leeuwis J.W., Broekhuizen R., Nguyen T.Q., de Borst G.J., Nathoe H.M., Verhaar M.C., Kok R.J. (2016). Plasma CTGF is independently related to an increased risk of cardiovascular events and mortality in patients with atherosclerotic disease: The SMART study. Growth Factors (Chur Switz.).

[18] Jaffa A.A., Usinger W.R., McHenry M.B., Jaffa M.A., Lipstiz S.R., Lackland D., Lopes-Virella M., Luttrell L.M., Wilson P.W. (2008). Connective tissue growth factor and susceptibility to renal and vascular disease risk in type 1 diabetes. J. Clin. Endocrinol. Metab..

[19] Nguyen T.Q., Tarnow L., Jorsal A., Oliver N., Roestenberg P., Ito Y., Parving H.H., Rossing P., van Nieuwenhoven F.A., Goldschmeding R. (2008). Plasma connective tissue growth factor is an independent predictor of end-stage renal disease and mortality in type 1 diabetic nephropathy. Diabetes Care.

[20] Cheung A.K., Rocco M.V., Yan G., Leypoldt J.K., Levin N.W., Greene T., Agodoa L., Bailey J., Beck G.J., Clark W. (2006). Serum beta-2 microglobulin levels predict mortality in dialysis patients: Results of the HEMO study. J. Am. Soc. Nephrol. JASN.

[21] Wang A.Y., Lai K.N. (2006). The importance of residual renal function in dialysis patients. Kidney Int..

[22] London G.M. (2003). Cardiovascular calcifications in uremic patients: Clinical impact on cardiovascular function. J. Am. Soc. Nephrol. JASN.

[23] Pecoits-Filho R., Barany P., Lindholm B., Heimburger O., Stenvinkel P. (2002). Interleukin-6 is an independent predictor of mortality in patients starting dialysis treatment. Nephrol. Dial. Transplant. Off. Publ. Eur. Dial. Transpl. Assoc. Eur. Ren. Assoc..

[24] Roestenberg P., van Nieuwenhoven F.A., Wieten L., Boer P., Diekman T., Tiller A.M., Wiersinga W.M., Oliver N., Usinger W., Weitz S. (2004). Connective tissue growth factor is increased in plasma of type 1 diabetic patients with nephropathy. Diabetes Care.

[25] Ito Y., Aten J., Nguyen T.Q., Joles J.A., Matsuo S., Weening J.J., Goldschmeding R. (2011). Involvement of connective tissue growth factor in human and experimental hypertensive nephrosclerosis. Nephron. Exp. Nephrol..

[26] Cozzolino M., Biondi M.L., Banfi E., Riser B.L., Mehmeti F., Cusi D., Gallieni M. (2010). CCN2 (CTGF) gene polymorphism is a novel prognostic risk factor for cardiovascular outcomes in hemodialysis patients. Blood Purif..

[27] Yao Y., Li B., Fu C., Teng G., Ma G., Liu N. (2017). Anti-connective tissue growth factor detects and reduces plaque inflammation in early-stage carotid atherosclerotic lesions. Nanomed. Nanotechnol. Biol. Med..

[28] Sanchez-Lopez E., Rayego S., Rodrigues-Diez R., Rodriguez J.S., Rodrigues-Diez R., Rodriguez-Vita J., Carvajal G., Aroeira L.S., Selgas R., Mezzano S.A. (2009). CTGF promotes inflammatory cell infiltration of the renal interstitium by activating NF-kappaB. J. Am. Soc. Nephrol. JASN.

[29] Arizono K., Nomura K., Motoyama T., Matsushita Y., Matsuoka K., Miyazu R., Takeshita H., Fukui H. (2004). Use of ultrapure dialysate in reduction of chronic inflammation during hemodialysis. Blood Purif..

[30] Furuya R., Kumagai H., Takahashi M., Sano K., Hishida A. (2005). Ultrapure dialysate reduces plasma levels of beta2-microglobulin and pentosidine in hemodialysis patients. Blood Purif..

[31] Weng L., Wang W., Su X., Huang Y., Su L., Liu M., Sun Y., Yang B., Zhou H. (2015). The Effect of cAMP-PKA Activation on TGF-beta1-Induced Profibrotic Signaling. Cell. Physiol. Biochem. Int. J. Exp. Cell. Physiol. Biochem. Pharmacol..

[32] Happe H., van der Wal A.M., Leonhard W.N., Kunnen S.J., Breuning M.H., de Heer E., Peters D.J. (2011). Altered Hippo signalling in polycystic kidney disease. J. Pathol..

[33] Attanasio M., Uhlenhaut N.H., Sousa V.H., O’Toole J.F., Otto E., Anlag K., Klugmann C., Treier A.C., Helou J., Sayer J.A. (2007). Loss of GLIS2 causes nephronophthisis in humans and mice by increased apoptosis and fibrosis. Nat. Genet..

[34] Hassane S., Leonhard W.N., van der Wal A., Hawinkels L.J., Lantinga-van Leeuwen I.S., ten Dijke P., Breuning M.H., de Heer E., Peters D.J. (2010). Elevated TGFbeta-Smad signalling in experimental Pkd1 models and human patients with polycystic kidney disease. J. Pathol..

[35] Norman J. (2011). Fibrosis and progression of autosomal dominant polycystic kidney disease (ADPKD). Biochim. Et Biophys. Acta.

[36] Gauer S., Holzmann Y., Kranzlin B., Hoffmann S.C., Gretz N., Hauser I.A., Goppelt-Struebe M., Geiger H., Obermuller N. (2017). CTGF Is Expressed During Cystic Remodeling in the PKD/Mhm (cy/+) Rat Model for Autosomal-Dominant Polycystic Kidney Disease (ADPKD). J. Histochem. Cytochem. Off. J. Histochem. Soc..

[37] Ito Y., Aten J., Bende R.J., Oemar B.S., Rabelink T.J., Weening J.J., Goldschmeding R. (1998). Expression of connective tissue growth factor in human renal fibrosis. Kidney Int..

[38] Leypoldt J.K., Cheung A.K., Deeter R.B. (1999). Rebound kinetics of beta2-microglobulin after hemodialysis. Kidney Int..

[39] Grooteman M.P., van den Dorpel M.A., Bots M.L., Penne E.L., van der Weerd N.C., Mazairac A.H., den Hoedt C.H., van der Tweel I., Levesque R., Nube M.J. (2012). Effect of online hemodiafiltration on all-cause mortality and cardiovascular outcomes. J. Am. Soc. Nephrol. JASN.

[40] Ward R.A., Greene T., Hartmann B., Samtleben W. (2006). Resistance to intercompartmental mass transfer limits beta2-microglobulin removal by post-dilution hemodiafiltration. Kidney Int..

[41] Grotendorst G.R., Duncan M.R. (2005). Individual domains of connective tissue growth factor regulate fibroblast proliferation and myofibroblast differentiation. FASEB J. Off. Publ. Fed. Am. Soc. Exp. Biol..

[42] Penne E.L., Blankestijn P.J., Bots M.L., van den Dorpel M.A., Grooteman M.P., Nube M.J., van der Tweel I., Ter Wee P.M. (2005). Effect of increased convective clearance by on-line hemodiafiltration on all cause and cardiovascular mortality in chronic hemodialysis patients—The Dutch CONvective TRAnsport STudy (CONTRAST): Rationale and design of a randomised controlled trial [ISRCTN38365125]. Curr. Control. Trials Cardiovasc. Med..

[43] Association for the Advancement of Medical Instrumentation (2014). Quality of dialysis fluid for hemodialysis fluid and related therapies ANSI/AAMI 11663:2014.

[44] Daugirdas J.T. (1993). Second generation logarithmic estimates of single-pool variable volume Kt/V: An analysis of error. J. Am. Soc. Nephrol. JASN.

[45] Fouque D., Vennegoor M., ter Wee P., Wanner C., Basci A., Canaud B., Haage P., Konner K., Kooman J., Martin-Malo A. (2007). EBPG guideline on nutrition. Nephrol. Dial. Transplant. Off. Publ. Eur. Dial. Transpl. Assoc. Eur. Ren. Assoc..

